# A Case of Idiopathic Thoracic Spinal Subdural Hematoma With Bilateral Lower Limb Paralysis

**DOI:** 10.7759/cureus.16585

**Published:** 2021-07-23

**Authors:** Takashi Dezawa, Keiji Hasegawa, Shintarou Tsuge, Akito Wada, Hiroshi Takahashi

**Affiliations:** 1 Department of Orthopaedic Surgery, Toho University School of Medicine, Tokyo, JPN

**Keywords:** idiopathic spinal subdural hematoma, paraplegia, laparoscopic surgery, bilateral lower limb paralysis, a posterior approach

## Abstract

Subdural hemorrhage is rare and is mostly triggered non-traumatically due to intracranial surgery, lumbar puncture, anticoagulant therapy, blood diseases, vascular malformations, and tumors. We experienced a case of idiopathic subdural hematoma with acute bilateral lower limb paralysis on postoperative day 4 after laparoscopic hysteromyomectomy.

The patient was a 40-year-old woman with uterine myoma who took no medication nor had history of trauma or abnormalities such as vascular malformations. Four days after laparoscopic surgery, sudden chest and back pain and bilateral lower limb paralysis appeared. Muscle weakness was found predominantly on the left side. In manual muscle test (MMT), the quadriceps femoris was 5/5 on both sides, but the tibialis anterior, extensor pollicis longus and flexor pollicis longus were 5/5 only on the right, and 2/5 on the left side. Warm pain sensation was decreased below Th4 (Fourth thoracic vertebra), and the right side showed a greater decrease of deep sensation than the left. Excretion was difficult and dysuria was also apparent. There were no abnormalities in blood biochemical tests or in the blood coagulation system. Using the results of diagnostic imaging, as preoperative diagnosis of the patient, spinal subdural hematoma was suspected. Conservative treatment was initially conducted but the emergency surgery for hematoma removal was performed at 14 hours after the onset because of progression of paralysis. This resulted in improvement of neurological symptoms including lower limb paralysis, bladder-rectal disorder and hypoesthesia.

If subdural hematoma is suspected regardless of the cause, it is important to observe neurological findings over time and make a quick decision to treat with surgery.

## Introduction

Hemorrhagic diseases in the spinal canal are roughly classified into intraspinal, epidural, subdural, and spinal subarachnoid hemorrhage. Subdural hemorrhage is rare, and most subdural hematomas are triggered non-traumatically due to intracranial surgery, lumbar puncture, anticoagulant therapy, blood diseases, vascular malformations, and tumors. Idiopathic cases triggered by unknown causes have also been reported [1,2]. Here, we report a case of idiopathic subdural hematoma with development of acute bilateral lower limb paralysis.

## Case presentation

The patient was a 40-year-old woman with myoma of the uterus. She was 172 cm tall and weighed 60 kg. She was not taking medication and her family history was unremarkable. She did not smoke, but she had a habit of consuming a small amount of alcohol a day. Laparoscopic hysteromyomectomy was performed under general anesthesia for treatment of uterine myoma. In laparoscopic surgery, we took pneumoperitoneum at 15 to 20 mmHg for one hour. Around 2:00 am on post operative day (POD) 4, sudden chest and back pain and bilateral lower limb paralysis appeared. The discharge schedule was canceled because it became impossible for the patient to stand on both sides. The obstetrics and gynecology on-duty doctor then requested our department to perform a closer examination.

At the time of the examination, chest and back pain had alleviated, and motor paralysis was also improving, with the patient able to kneel on the right side to an extent. However, she was unable to kneel on the left side and her manual muscle test (MMT) score was 2/5. About 1 hour later, headache appeared and a head CT scan was performed. This showed no abnormal findings, and headache disappeared after administration of acetaminophen. There were no abnormal neurological findings in the upper limbs, but increased deep tendon reflexes, hypoesthesia and muscle weakness were observed in both legs. These findings led to suspicion of thoracic spine disease. Since it was nighttime and paralysis seemed to be improving, cervical and thoracic spine MRI was delayed until the morning. This showed an abnormal finding from the cervical vertebra to the upper thoracic vertebra, and the patient was then formally transferred to the orthopedic department.

Upon transfer, her consciousness was clear and blood pressure was 139/93 mmHg. The electrocardiogram showed sinus rhythm with no arrhythmia, and there was no abnormality in respiratory spirometry. No abnormalities were found in deep tendon reflexes of both upper limbs, but bilateral patellar tendon and Achilles tendon reflexes were enhanced. Muscle weakness was found predominantly on the left side. In MMT, the quadriceps femoris was 5/5 on both sides, but the tibialis anterior, extensor pollicis longus and flexor pollicis longus were 5/5 only on the right, and 2/5 on the left side. Warm pain sensation was decreased below Th4, and the right side showed a greater decrease of deep sensation than the left. The temperature pain sensation was lost below the right knee and had almost disappeared below the left knee. Poor urination and dysuria were also apparent. She couldn't feel the urge to urinate and defecate and was incontinent. So a bladder catheter was inserted into her. There were no abnormalities in blood biochemical tests or in the blood coagulation system.

**Figure 1:**
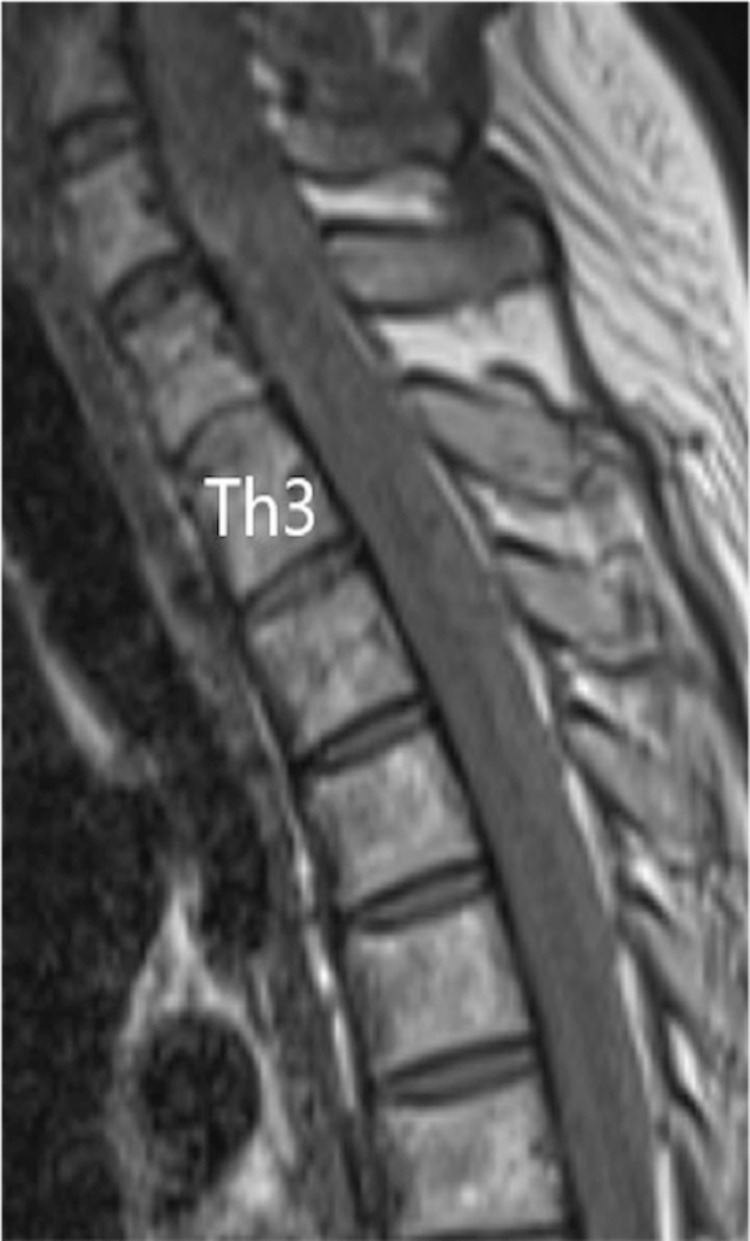
Sagittal T1-weighted MR images at the time of transfer A zonal change with a septum was observed on the right dorsal side of the spinal cord dura mater at the position from Th1 to Th6 whose T1-weighted image exhibited the signal equal to that of the spinal cord and higher than that of the cerebrospinal fluid.

**Figure 2:**
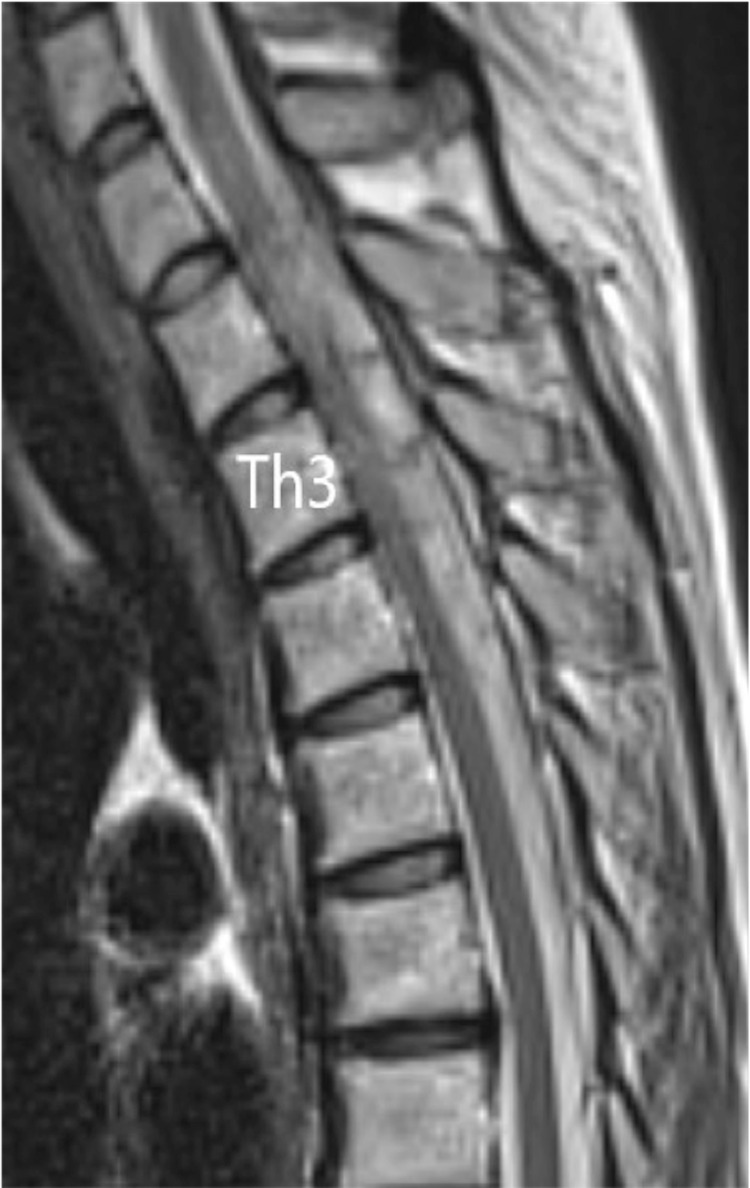
Sagittal T2-weighted MR images at the time of transfer In the T2-weighted image, the signal was higher than that of the spinal cord and lower than that of the cerebrospinal fluid.

**Figure 3:**
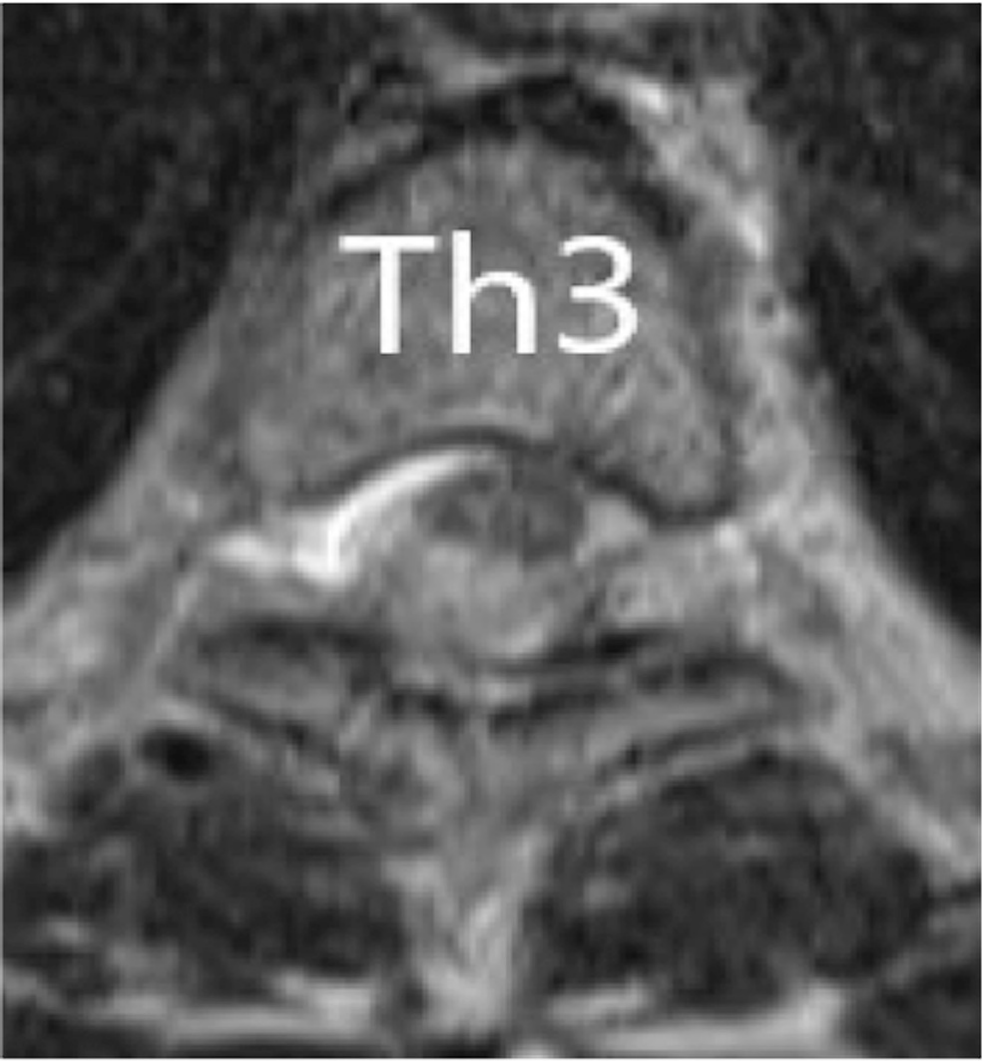
Axial T1-weighted MR images at the time of transfer The stenosis was located at the highest position of Th3, and the thoracic spinal cord was compressed to the left ventral side, with moderate to severe spinal cord compression.

A chest X-ray showed no clear enlargement of the upper mediastinum. On CT of the thoracic spine, a high-intensity region was found on the right dorsal side in the dura in the upper part of Th1 to Th6. In sagittal thoracic MRI, there was a zonal change with a septum on the right dorsal side of the spinal cord dura mater from Th1 to Th6. In this region, the signal intensity on a T1-weighted image was equal to that of the spinal cord and higher than that of the cerebrospinal fluid (CSF) (Figure 1), and on a T2-weighted image the intensity was higher than that of the spinal cord and lower than that of the CSF (Figure 2). A stenosis was located at an upper Th3 level, and the thoracic spinal cord was compressed to the left ventral side, with moderate to severe spinal cord compression (Figure 3).

After transfer to the orthopedic department, we decided to perform conservative treatment if symptoms improved, and surgery if progression was observed. Three hours after the first examination, there was no significant change in MMT right 5/5 and left 2/5 below the tibialis anterior muscle, and no change in paresthesia. Six hours later, there was no change in sensory deficits. However, lower limb MMT had decreased to 3/5 on the right side below the tibialis anterior muscle, and the left was still 2/5. This progression of bilateral lower limb paralysis led to the decision to perform emergency surgery 14 hours after the onset of symptoms.

**Figure 4:**
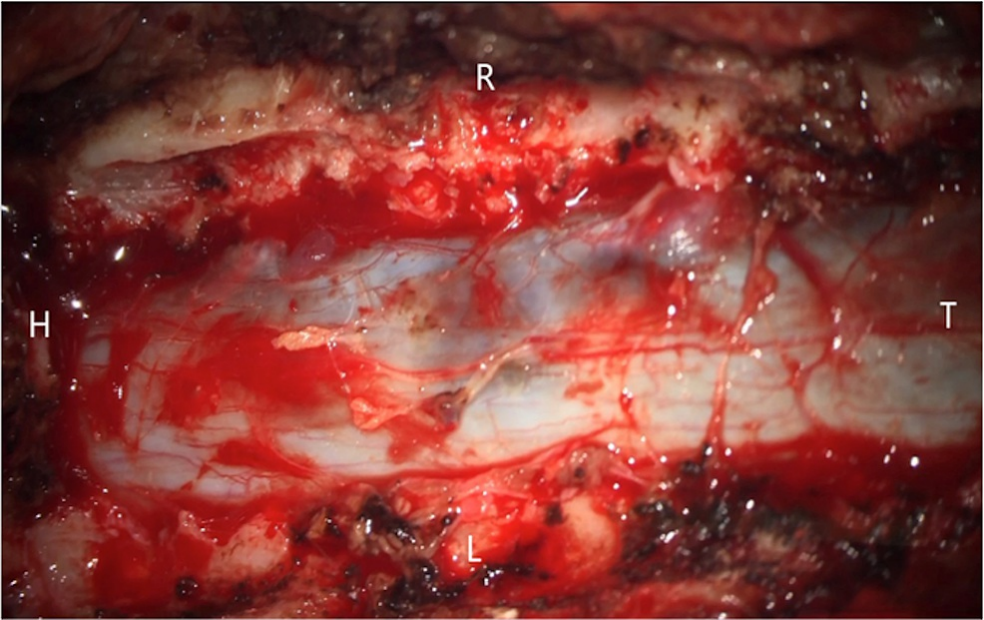
Surgical findings before dural incision A dark hematoma was seen under the dura at the level of the Th3 pedicle.

**Figure 5:**
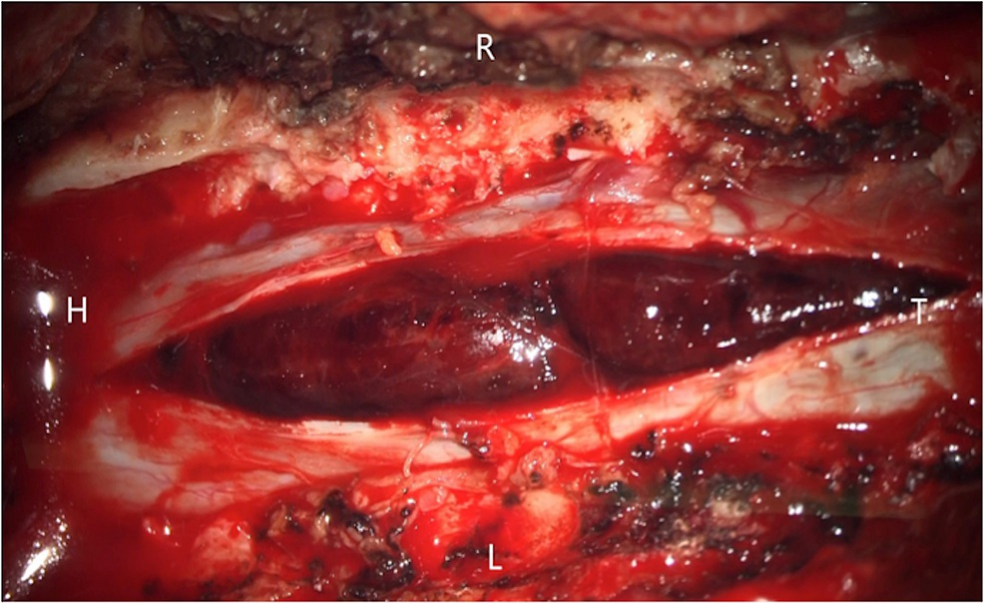
Surgical findings after dural incision After incising the dura, the hematoma was observed in the subarachnoid space.

A posterior approach was used to open Th1-5, and the Th2, Th3, and Th4 laminae were excised. Bleeding occurred from the dilated epidural venous plexus, but there was no epidural hematoma. A dark hematoma was seen under the dura at the level of the Th3 pedicle, with a bulge of the dura observed at the same site (Figure 4). Incision of the dura revealed a hematoma with a subdural coating (Figure 5). The hematoma was located in the right upper part of Th3, especially around the Th3 nerve root, and was mainly in the subarachnoid space. Hematoma at the Th2 to Th4 levels was removed by suction to the extent possible, and the dura was displaced to the ventral side and flattened. No clear bleeding source was found in or around the Th3 nerve root, and there was no tumor or arteriovenous malformation.

**Figure 6:**
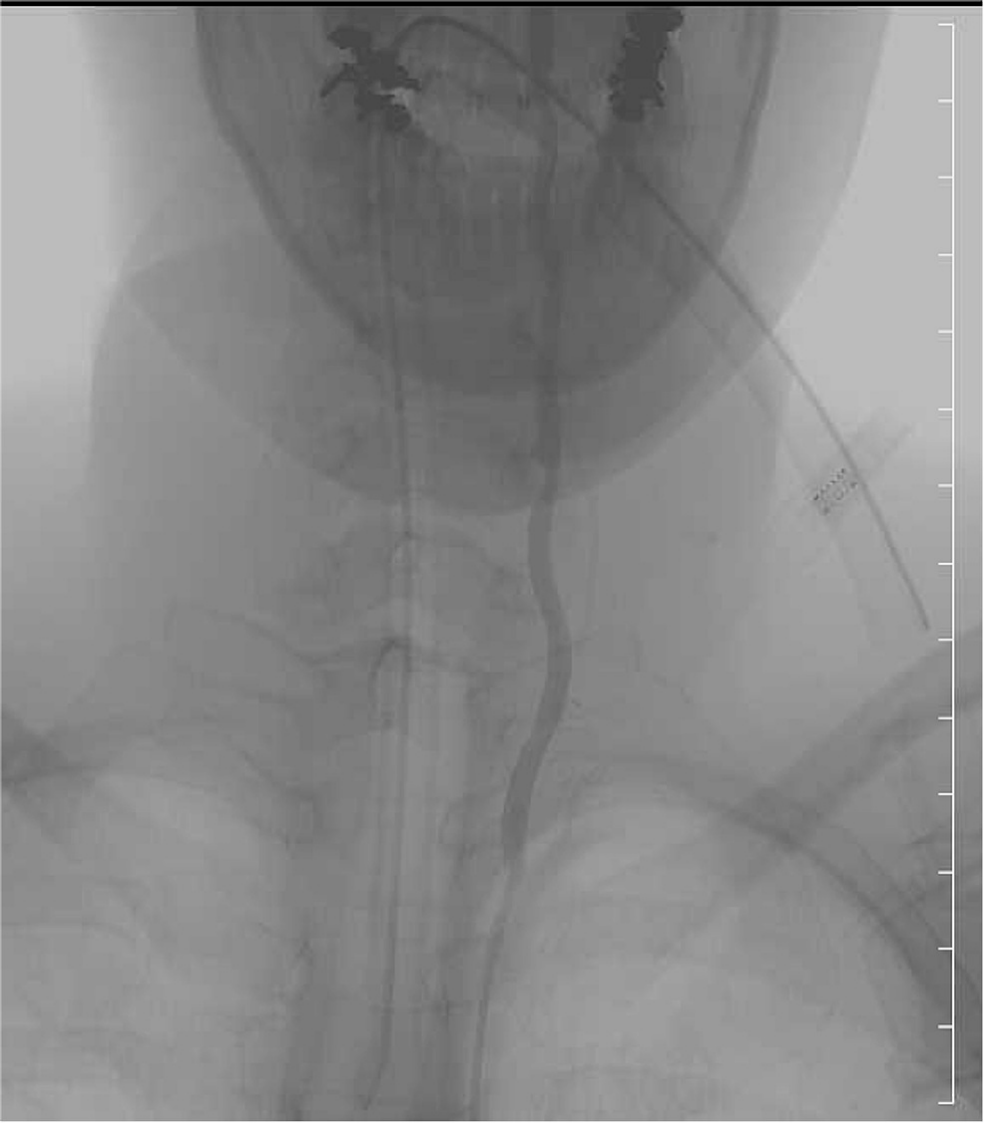
Selective spinal angiography, left common carotid artery No obvious abnormalities such as vascular malformations were observed.

**Figure 7:**
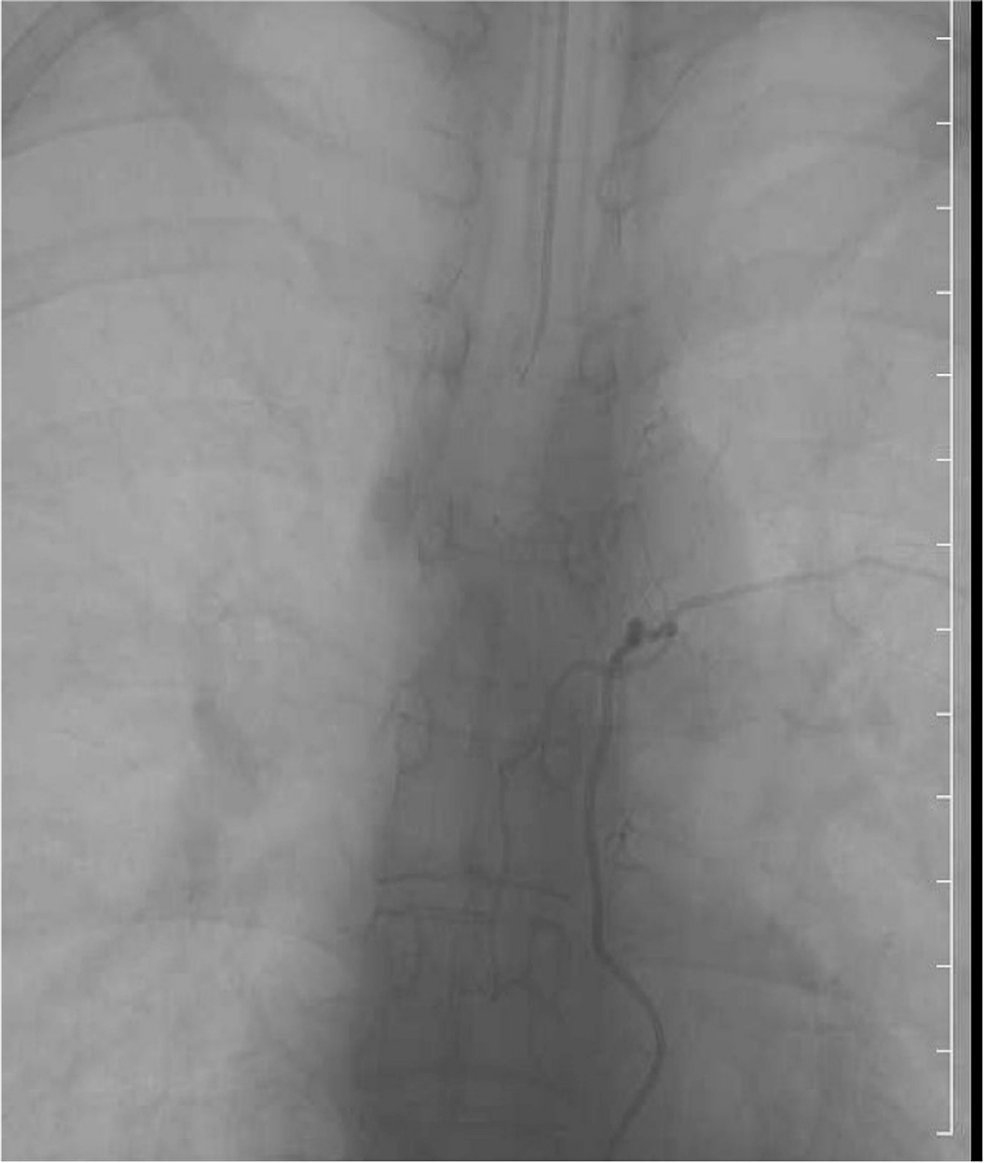
Selective spinal angiography, left fifth intercostal artery No obvious abnormalities such as vascular malformations were observed.

Immediately after the operation, sensation improved in the trunk and thighs. However, warm pain sensation was lost in the area below the right L5 region. MMT for below the tibialis anterior muscle was 3/5 on the right and 2/5 on the left. Moreover, excretion was difficult, although there was an urge to urinate and a bowel movement. Selective spinal angiography was performed one week after surgery (Figures 6, 7), but there were no obvious abnormalities such as vascular malformations. Four weeks after the operation, MRI of the thoracic spine showed disappearance of the hematoma, but there was a high-intensity region on the left side of the spinal cord at the Th3 level, accompanied by atrophy. In addition, T2-weighted images of the lower thoracic cord to the conical spinal cord area showed a low signal, suggesting hemosiderin deposition.

**Figure 8:**
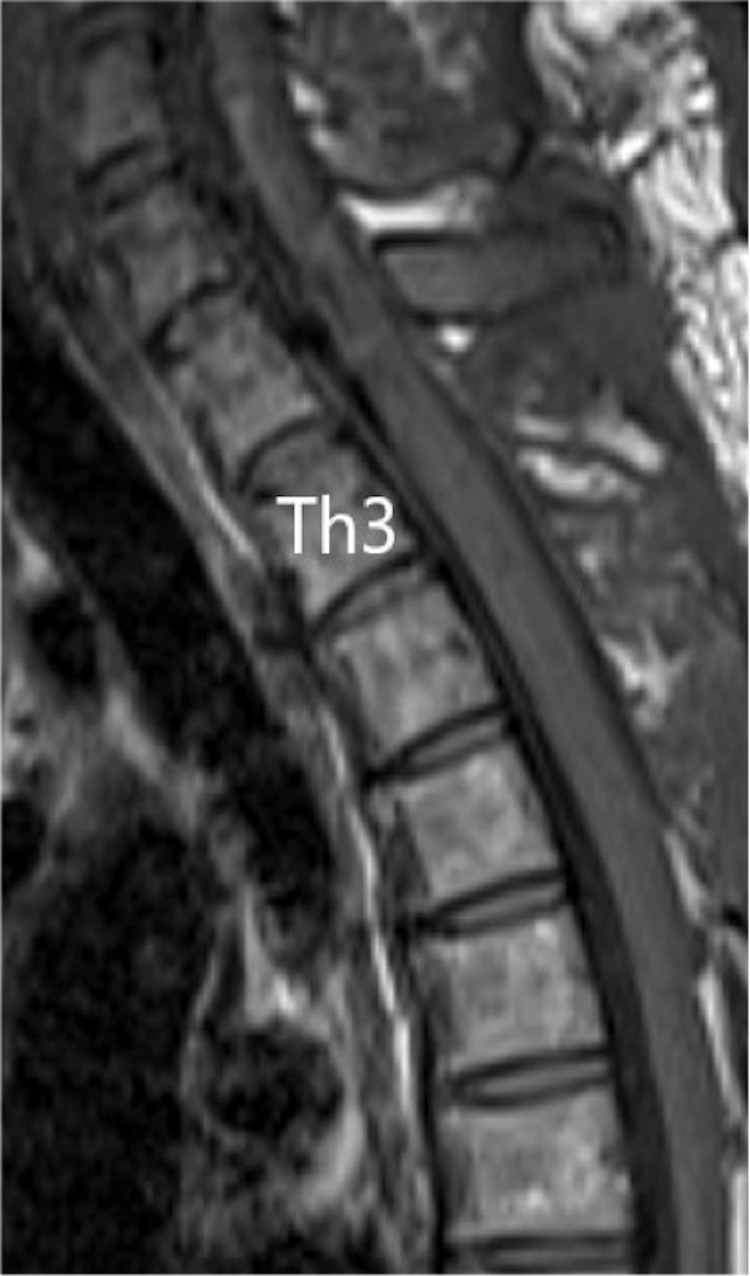
Sagittal T1-weighted MR images 12 weeks after the operation

**Figure 9:**
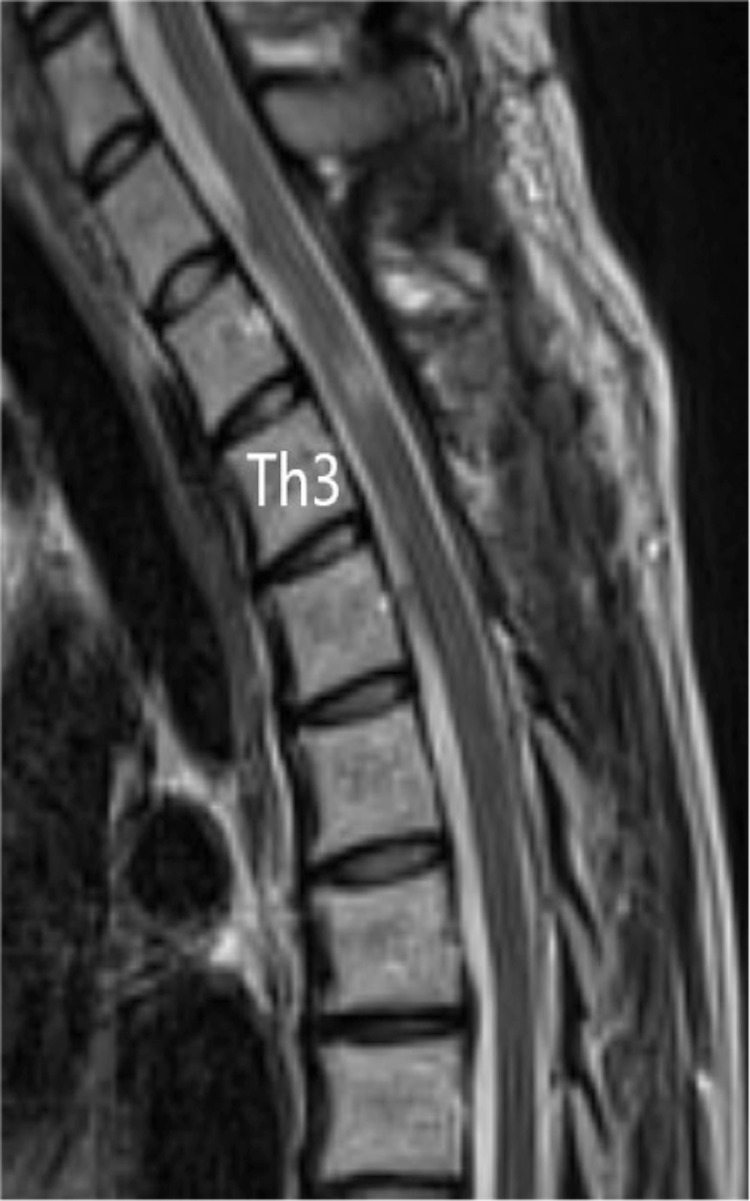
Sagittal T2-weighted MR images 12 weeks after the operation The high-intensity region in the spinal cord of the Th2-weighted image at the higher position of Th3 remained, but the hematoma disappeared.

**Figure 10:**
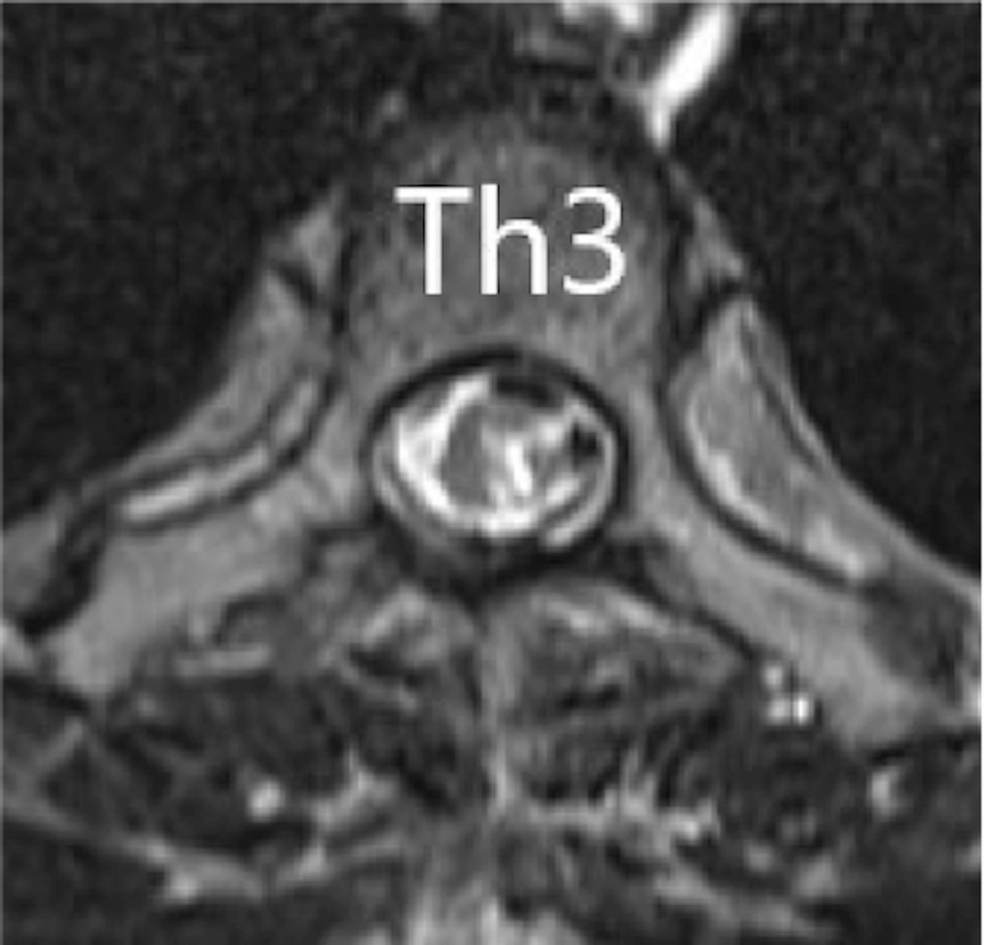
Axial T2-weighted MR images 12 weeks after the operation

Paralysis of both lower limbs recovered over time, and the patient was able to walk with a brace on her left ankle four weeks after surgery, and to walk independently five weeks after onset. Defecation improved to a controllable level, but urination was not possible, despite awareness of the need to urinate. For this reason, a urethral catheter was placed. The patient was discharged to a convalescent rehabilitation hospital six weeks after onset with independent walking. At 12 weeks postoperatively, the patient was able to walk independently and MMT below the quadriceps femoris was 5/5 on the right and left sides. Deep sensation was slightly reduced on the left, but improved overall. The patient was able to urinate, but with slight decrease in urgency due to bladder-rectal disorder. Thoracic spine MRI of the spinal cord showed a high-intensity region at the upper Th3 level on a T2-weighted image, but the hematoma had disappeared (Figures 8-10).

## Discussion

Subdural hematoma is a rare disease because the spinal canal is protected by vertebrae and paraspinal muscles, and unlike the epidural space, there is no major blood vessel or bridging vein in the subdural space [3]. Iatrogenic lumbar puncture and epidural anesthesia are known causes of onset, and there are several reports of cases of bleeding diathesis caused by a combination of anticoagulant therapy and thrombocytopenia. Bleeding due to rupture of a spinal arteriovenous malformation or hemangioma, other causes, and idiopathic cases have also been reported [1,2]. Subdural hematoma in elderly patients often accompanies administration of antiplatelet agents, whereas cases in younger patients tend to be caused by vascular malformations or bleeding from hemangiomas. In our case, the patient had no history of oral administration of anticoagulants or antithrombotics, and no arteriovenous malformations or tumors were found by selective spinal angiography. No history of trauma or falls was observed within two to three weeks before the appearance of symptoms.

Possible cause is that post-surgery pain may have increased blood pressure, resulting in the collapse of blood vessels in the spinal cord. The radicular vein in the subarachnoid space may also have contributed to the rupture, but there was no abnormality immediately after the operation. We also confirmed retrospectively that intraoperative and postoperative blood pressure did not show substantial changes, and this was also unlikely to be a cause because onset occurred on POD 4.

The two major bleeding sites are the subarachnoid and subdural spaces [4,5]. In a case of hemorrhage from the subarachnoid space, the radicular vein, which runs through the subarachnoid space and has no valves, is thought to rupture due to an increase in internal pressure in the thoracic or abdominal cavity or a slight trauma. The resulting hemorrhage may then break the arachnoid and form a subdural hematoma. Hemorrhage from the subdural space is thought to be caused by bleeding from the fine and extradural blood vessels on the inner surface of the dura. At the same time, hemorrhage of the subarachnoid space reaches the bleeding subdural space by breaking the arachnoid membrane [4,5].

In the current case, continuous hematomas were present in the subdural and subarachnoid spaces, as observed after an incision of the dura and arachnoid. Observation of a small amount of CSF suggested that the arachnoid membrane was damaged. No clear bleeding site could be identified, but the presence of a large hematoma around the right Th3 nerve root and the intraoperative findings of a larger hematoma in the subarachnoid space strongly suggested bleeding from the subarachnoid space.

Intrathecal hematoma can be divided into subarachnoid, subdural, and epidural hematoma, but all have sudden onset of lumbar back pain, followed by motor palsy, sensory deficits, and autonomic neuropathy that appear over time. Differential diagnosis is often based on imaging due to the similar clinical symptoms, and our case was diagnosed as subdural hemorrhage using MRI. Unlike epidural hematoma, subarachnoid hematoma is characterized by a liquid surface image. Subdural hematomas also often occur from the lumbar spine to the thoracic level, with extension craniocaudally from 1 to 19 vertebral bodies, and on the ventral and dorsal sides of the spinal cord, but slightly on the ventral side. In contrast, epidural hematomas are more often found from the cervical vertebra to the upper thoracic level, and are usually localized between 2 and 3 vertebral bodies, often on the dorsal side.

Subdural hematomas can be diagnosed based on the crescent shape that covers the spinal cord. In contrast, an epidural hematoma has a convex shape and does not spread, unlike a subdural hematoma. The dura appears as a low-signal structure that borders the hematoma and spinal cord in epidural hematomas, but not in subdural hematomas. Furthermore, epidural fat often disappears due to being covered by an epidural hematoma, whereas with subdural hematomas, the epidural fat is visualized farther from the hematoma than the dura. MRI in our case showed hematomas with a zonal change with a capsule and a lower intensity signal than that of CSF on the dorsal side of the dura, and a high-intensity signal area in a T1-weighted image that was considered to be epidural fat [6,7]. These findings clearly supported diagnosis of a subdural hematoma, rather than an epidural hematoma. In imaging, subdural and subarachnoid hematomas can be distinguished by the presence of spinal fluid showing a subarachnoid space between the hematoma and spinal cord [8]. In our case, intraoperative findings were associated with subdural and subarachnoid hematomas, and it was difficult to distinguish these hematomas on MRI.

Treatment of idiopathic spinal subdural hematoma is divided into conservative therapy and surgery. Some reported cases showed improvement of symptoms with conservative therapy alone, and in others steroid pulse therapy was effective [9,10]. After hematoma removal by surgery, both good and poor postoperative courses have been reported, and surgery does not always lead to good recovery of symptoms. Thus, there is no consensus on treatment of acute subdural hematoma, but surgery is recommended if there is aggravation of neurological findings such as paralysis. The treatment outcome depends on the size of the hematoma, degree of paralysis, progression, and timing of surgery [11-14]. In our case, hematoma removal surgery was performed 14 hours after the appearance of symptoms, and this resulted in improvement of neurological symptoms such as bladder-rectal disorder, paralysis and hypoesthesia.

## Conclusions

We encountered a case of idiopathic spinal subdural hematoma that developed on POD 4 after laparoscopic surgery with symptoms of sudden back pain and paralysis of both legs, and was cured by surgery. Regardless of the cause, if subdural hematoma is suspected it is important to observe neurological findings over time and make a quick decision to treat with surgery.
